# Functional Ability Improved in Essential Tremor by IncobotulinumtoxinA Injections Using Kinematically Determined Biomechanical Patterns – A New Future

**DOI:** 10.1371/journal.pone.0153739

**Published:** 2016-04-21

**Authors:** Olivia Samotus, Fariborz Rahimi, Jack Lee, Mandar Jog

**Affiliations:** 1 Department of Clinical Neurological Sciences, London Health Sciences Centre–Lawson Health Research Institute, London, Ontario, Canada; 2 Schulich School of Medicine and Dentistry, Western University, London, Ontario, Canada; 3 Department of Electrical and Computer Engineering, University of Bonab, Bonab, East Azerbaijan, Iran; University of Toronto, CANADA

## Abstract

**Objective:**

Effective treatment for functional disability caused by essential tremor is a significant unmet need faced by many clinicians today. Current literature regarding focal therapy by botulinum toxin type A (BoNT-A) injections uses fixed dosing regimens, which cannot be individualized, provides only limited functional benefit and unacceptable muscle weakness commonly occurs. This 38-week open label study, the longest to-date, demonstrates how kinematic technology addressed all these issues by guiding muscle selection.

**Method:**

Participants (n = 24) were assessed at weeks 0, 6, 16, 22, 32, and 38 and injected with incobotulinumtoxinA at weeks 0, 16, and 32. Clinical assessments including UPDRS tremor items, Fahn-Tolosa-Marin (FTM) tremor rating scale assessing tremor severity, writing and functional ability, quality of life questionnaire (QUEST) and objective kinematic assessments were completed at every visit. Participants performed two postural and two weight-bearing scripted tasks with motion sensors placed over the wrist, elbow and shoulder joints. These sensors captured angular tremor amplitude (RMS units) and acceleration joint motion that was segmented into directional components: flexion-extension (F/E), pronation-supination and radial-ulnar at the wrist, F/E at the elbow, and F/E and adduction-abduction at the shoulder. Injection parameters were determined using kinematics, followed by the clinician’s determination of which muscles would contribute to the specific upper limb tremor biomechanics and dosing per participant.

**Results:**

Multi-joint biomechanical recordings allowed individualized muscle selection and showed significant improvement in whole-arm function, FTM parts A-C scores, at week 6 which continued throughout the study. By week 38, the total FTM score statistically significantly reduced from 16.2±4.6 at week 0 to 9.5±6.3 (p<0.0005). UPDRS item 21 score rating action tremor was significantly reduced from 2.6±0.5 at week 0 to 1.6±1.1 (p = 0.01) at week 32. Quality of life (QUEST) significantly improved from 40.3±15.8 at week 0 to 31.1±15.3 (p = 0.035) at week 32 and to 27.8±15.3 (p = 0.028) at week 38. Kinematics provided an objective, secondary outcome measure, which showed a significant decrease in tremor amplitude in the wrist and shoulder joints (p<0.05). Eight participants (40%) self-reported mild weakness in injected muscles but had no interference in arm function.

**Conclusion:**

Kinematic tremor assessments provide the injector unique insight to objectively individualize and personalize injection parameters demonstrating BoNT-A effectively alleviates functional disability caused by essential tremor. Kinematic technology is a promising method for standardizing assessments and for focal upper limb tremor treatment.

**Trial Registration:**

ClinicalTrials.gov NCT02427646

## Introduction

One of the most prevalent movement disorders is essential tremor (ET), affecting 4.6% of people aged 65 and older [1]. ET is visually characterized by persistent, bilateral, mainly symmetrical postural or kinetic tremor involving distal and/or proximal arm muscles. The severity of ET gradually increases over time that may cause significant difficulty with daily tasks such as eating, grooming and other fine motor tasks. Functional disability due to tremor greatly affects the quality of life in patients who subsequently seek treatment. The most effective oral medications to symptomatically treat mild or moderate ET are primidone, an anticonvulsant, and propranolol, a beta-adrenergic receptor antagonist [2]. Although these agents reduce tremor amplitude by approximately 50%, they provide limited functional benefit and adverse side effects such as dizziness, fatigue, and bradycardia commonly occur [3]. In addition, 30% of patients have no therapeutic benefit leaving a large population with severe ET untreated [4]. Surgical therapy by thalamotomy or by unilateral/bilateral thalamic deep brain stimulation is safe, although possibly effective (Level C recommendation) and is performed in patients under the age of 75 where post-operative device programming remains unclear [2,5]. Thus, a new approach for treating debilitating tremor is still a significant unmet need.

Botulinum neurotoxin type A (BoNT-A) intramuscular injections are commonly used to treat various movement disorders, such as focal dystonias, and may provide modest beneficial effects in essential tremor patients who are unresponsive to conventional pharmacotherapies [6,7]. Prior studies have reported that BoNT-A therapy reduces the severity of postural tremor with minimal improvements in clinical scores [8–10]. Despite this modest clinical benefit, BoNT-A therapy has not been widely adopted due to risk of significant hand and wrist weakness causing functional limb impairment [7]. The primary drawback of these prior studies was utilizing a rigid dosing regimen which did not individualize and target appropriate muscles nor were appropriate BoNT-A dosages applied which failed to provide functional benefit. The fixed dosing regimen considers that tremor is similar across patients and across joints thereby defeats the advantage of using BoNT-A as a targeted and individualized focal therapy. The variation in tremor dynamics is considerable and multiple joints, wrist, elbow and shoulder, can be involved to differing degrees making visual assessments a significant challenge. Hence, it is clear that proper identification of the dynamics of the tremulous joints would allow individualization of muscle selection necessary to optimize injection pattern and outcomes.

Subjective tremor assessment tools, such as clinical rating scales are inaccurate methods of clinical evaluation that are not specific to tremor type. Characterization of tremor by kinematic methodology is objective and superior to visual inspection alone, a challenging task for a clinician. Preliminary work shows that identification of tremulous joints and muscles by accelerometers and by surface EMG can reduce the occurrence of muscle weakness [11]. Minimization of dose-dependent weakness is achievable by utilizing such objective methods to deliver individualized injection patterns [12,13].

Multi-sensor technology has been well established, is becoming very affordable and is capable of characterizing tremor at the wrist, elbow, and shoulder [14,15]. Objective measures of the severity and direction of tremulous movements along the whole limb can be used for selecting injection sites and BoNT-A dose per muscle. Recent work in treatment of Parkinson disease (PD) tremor using methodology discussed in this paper has been successful [16]. However, clinical and biomechanical features of PD tremor are distinctly different from those seen in ET. In addition, the postural and kinetic nature of ET tremor results in different joint biomechanics and the individualization of injection patterns based upon kinematics are considerably different than in PD tremor. The current paper shows results of a first of its kind study use a kinematically guided, individualized, multi joint upper limb injection approach to determine the efficacy and safety of incobotulinumtoxinA as a focal treatment in the longest, 38-week duration open label study involving ET participants.

## Methods

### Study Timeline

This single-centre, single-injector, open label (Health Canada CTA# 178589) pilot study recruited a convenience sampling of 24 ET participants from the London Movement Disorder Centre who completed six study visits at weeks 0, 6, 16, 22, 32, and 38 and were injected at weeks 0, 16, and 32. Assessments were carried out at all visits and peak dose effects were measured at 6 weeks after each visit. As prior studies have stated that BoNT-A peak effect is evident approximately 4 weeks to 8 weeks post-injection, the designated time-point for a follow-up visit occurred 6 weeks post-injection [17]. Treatments were administered every 4 months instead of every 3 months, which is typically followed in the clinic setting, to incorporate a BoNT-A washout period of 1 month [17]. Each study visit consisted of clinical scales and kinematic tremor measurements. Medication was not withheld from participants during study visits. The tremor dominant limb was injected with incobotulinumtoxinA (0.5 mL of saline per 100 unit vial) using needle electromyographic (EMG) guidance (1” long 30 g injectable EMG needle using a Clavis^®^ portable EMG machine). First participant first visit and last participant last visit occurred on January 2012 and April 2014, respectively.

### Study Criteria

**Fig 1:**
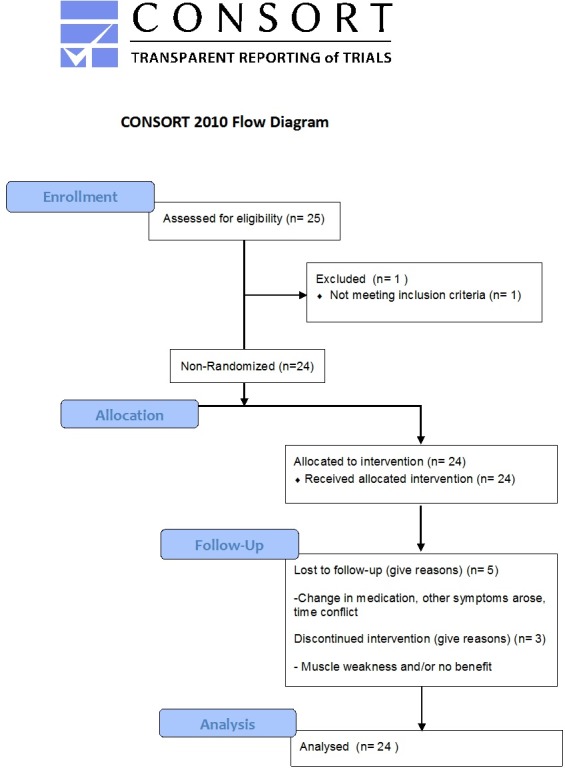
CONSORT flow diagram displaying the progress of the study’s design.

This study protocol was approved by Western University Health Sciences Research Ethics Board (REB#18445) on March 28, 2012 as a clinical phase IIb pilot study. The power calculation provided in the ethics study protocol submission suggested a target sample size of 35 ET participants, though this calculation was based on literature which did not incorporate kinematics or any objective data for guiding BoNT-A injections for tremor. As this is an open-label pilot study with no randomization, a convenience sampling was reported for those that were screened (n = 25) and for those that participated in the study (n = 24). Additionally, the duration of the study stated in the approved protocol is for a 96-week study over thirteen study visits. However, the current results were significant at the timeline reported in the manuscript (six study visits over 38 weeks) as serial BoNT-A for upper limb tremor have been sparsely reported in this manner. Participants provided written consent to participate in this study by signing the study’s consent form. The ethics committee provided full board approval for this study protocol and consent procedure was approved as required in the consent documentation checklist, submitted with the full study protocol. Registration with a clinical trial registry was not a requirement for ethics approval to perform the study at this institution. The authors confirm that all ongoing and related trials for this drug/intervention are now registered (ClinicalTrials.gov Identifier: NCT02427646). See Fig 1 for the CONSORT flowchart and the supporting information (S2 File) for the TREND statement checklist.

The inclusion criteria consisted of male and female participants, aged 18 to 80 years diagnosed with ET with upper limb tremor as their primary and most bothersome symptom for at least two years, incobotulinumtoxinA naïve, on stable medication management for a minimum of six months prior to study enrolment, with none withheld or adjusted during the study. At enrollment, participants were either stable on their anti-tremor medications, unable to tolerate oral medications, or unwilling to comply due to side effects. Exclusion criteria were those who had a history of stroke, contraindications per the incobotulinumtoxinA monograph, pregnancy, and existing pharmacological therapy with tremor-inducing side effects (e.g. lithium, valproate, steroids, amiodarone, or beta-adrenergic agonists such as salbutamol).

### Kinematic Assessment

Participants performed a series of scripted tasks (“posture-1” (arms outstretched, palms down), “posture-2” (palms facing inwards), “load-1” (empty cup), and “load-2” (cup with 1 lb. weight)) while seated, thrice in series for 20 seconds per trial (S1 Fig). Motion sensor devices were placed over each the forearm, wrist, elbow and shoulder joints S2 Fig) capturing tremor severity in angular RMS amplitude simultaneously in multiple degrees of freedom (DOF): flexion-extension (F/E), pronation-supination (P/S), and radial-ulnar (R/U) deviations in the wrist, F/E in the elbow, and F/E and abduction-adduction (Abd/Add) deviations in the shoulder. Accelerometers placed on the middle finger and on the hand captured tremor simultaneously with the motion sensors though accelerometry measures were not utilized for determining injection parameter. See supporting information for the detailed kinematic protocol.

### Injection Determination

Custom written software in MatLab^®^ (R2011a) processed raw angular signal data captured by the motion sensors [13,15,16]. The interpreted data displayed tremor severity, as total angular RMS amplitude, in each DOF during each task in each arm joint that was reviewed by a clinician prior to injection. The software provided a percentage contribution of the directional movements. Based upon the experienced clinician’s best judgment, a preselected total dose based on tremor amplitude was divided using the percentage contribution data and was allocated to appropriate muscles for injection. Muscles selected for injection were based upon well-known anatomical basis of movement at each joint. Dosages for subsequent injection visits were based upon comparisons of kinematics at that visit to prior kinematic data. This approach allowed the experienced clinician to use the kinematic data to tailor the injections at each joint and to ensure the most appropriate muscles were selected, making the approach generalizable in the experienced clinician’s hands.

### Clinical Scale Assessment

Validated tremor severity and functional rating scales were used as primary endpoints for measuring efficacy and safety of incobotulinumtoxinA treatments. Participants completed the Fahn-Tolosa-Marin (FTM) Scale consisting of parts A-C rating tremor severity, writing and functional disability caused by tremor, and the Quality of Life for Essential Tremor (QUEST) questionnaire encompassing 30-items rating physical, psychosocial, communication, hobbies/leisure, and work/finance, The response to the 30-items, ranging from never = 0, rarely = 1, sometimes = 2, frequently = 3, and always = 4, were tallied for each participant for each study visit. The assessor monitored strength in participant’s fingers, distal, and proximal arm muscles by manual muscle testing, and by maximal grip strength, using a dynamometer. Muscle weakness reported by participant was assessed using a Likert scale (ranging from 0 = no weakness to 4 = severe weakness in whole arm). The movement disorder neurologist, blinded to prior results, assessed tremor using UPDRS (items 20 and 21) during injection visits.

### Statistical Analyses

IBM^®^ SPSS^®^ statistics version 20 was used to analyze both kinematic and clinical data using one-way repeated measures analysis of variance (ANOVA) using confidence intervals of 95% (ɑ = 0.05) with post hoc Bonferroni corrections for multiple comparisons performed across all time-points. Missing of random value analysis was conducted for all independent variables to ensure incomplete data sets were missing completely at random. Participant clinical rating scores from each time-point were analyzed by mean and standard deviation of the population. The mean angular RMS tremor amplitude across three trials for each task per study visit was log-transformed as tremor amplitudes generated skewed distributions. Acceleration values were computed by averaging the acceleration in X-axis, Y-axis and Z-axis for each task per participant at a visit. Each task was performed thrice in series per kinematic recording session. The means from each clinical rating scale and from the kinematic tremor analyses that met criteria were tested for normality using the Shaprio-Wilk test and z-score for skewness and kurtosis. The means which met criteria for parametric analysis underwent parametric ANOVA tests to investigate the presence of significant changes between time-points. If the means did not meet criteria for parametric analysis, the Friedman ANOVA test was conducted. A p-value < 0.05 for the Mauchly’s test of sphericity indicated that the assumption of sphericity had been violated. The Greenhouse-Geisser correction of this bias was used when the estimated epsilon (ε) was less than 0.75 or by the Huynh-Feldt correction if estimated epsilon (ε) was greater than 0.75. Partial eta-squared (partial η^2^) was reported as an estimate of the population effect size.

## Results

### Participant Demographics

Demographics and baseline clinical rating scores of the 24 ET participants are summarized in Table 1. 25.0% of participants (6/24) were being treated with primidone (mean dose of 125 mg/day). 4.2% of participants (1/24) withdrew following week 22 due to a myocardial infarction, unrelated to the study intervention. At week 38, 4.2% of participants (1/24) were withdrawn due to failed attendance and 4.2% of participants (1/24) withdrew due to unwanted weakness.

**Table 1:** ET participant demographics and baseline UPDRS, QUEST and FTM parts A to C scores.

							Injected Limb								
ID	Sex	Age	Weight (lbs)	Medications^a^	Motor Dominant Limb	Injected Limb	QUEST Score	UPDRS Item 20 (/4)	UPDRS Item 21 (/4)	FTM Part A Rest Tremor (/4)	FTM Part A Postural Tremor (/4)	FTM Part A Action Tremor (/4)	FTM Part B Spirals (/8)	FTM Part B Lines (/4)	FTM Part C Functional Disability (/28)
**1**	M	76	175	Primidone (125 mg)	R	R	31	1	3	2	2	3	6	2	14
**2**	F	74	165	Primidone (125mg)	R	R	27	1	2	1	2	2	4	1	11
**3**	M	67	270	N/A	R	R	39	1	3	2	4	2	4	1	14
**4**	M	76	223	Primidone (125 mg)	R	R	49	2	2.5	1	3	3	6	2	20
**5**	M	78	220	Primidone (125mg)	R	R	27	0	3	0	3	3	2	1	17
**6**	M	84	225	Propranolol (180mg), primidone (250mg)	R	R	30	2	2.5	2	3	3	5	2	22
**7**	F	64	120	N/A	R	R	49	0	3.5	2	3	3	5	3	21
**8**	F	71	140	N/A	R	R	48	1	3.5	1	4	0	6	3	10
**9**	M	61	167	N/A	R	R	61	0	2.5	2	3	3	4	3	18
**10**	F	82	120	N/A	R	L	22	0	3	0	0	3	8	2	15
**11**	F	68	205	Quetiapine (400mg), Omeprazole (40mg),	L	L	49	0	3	3	3	3	7	4	17
**12**	M	85	221	N/A	R	R	5	0	2.5	3	2	2	5	1	14
**13**	F	51	160	N/A	R	R	40	1	2	1	1	0	3	2	13
**14**	F	66	300	N/A	R	R	61	0	3	1	3	1	7	3	23
**15**	F	78	155	N/A	R	R	47	0	2	2	2	1	2	2	14
**16**	F	65	270	N/A	R	R	42	0	3	1	2	1	4	2	12
**17**	M	80	175	N/A	R	R	76	0	3.5	0	3	2	8	4	29
**18**	F	80	130	N/A	R	R	64	1	2	1	2	1	2	2	17
**19**	M	61	270	N/A	R	R	44	0	2	2	2	1	2	1	13
**20**	F	73	200	N/A	R	R	22	0	2	2	2	1	2	1	12
**21**	M	84	175	N/A	R	R	39	0	2	0	1	2	8	1	20
**22**	M	59	227	N/A	R	R	31	0	2	1	1	1	2	1	11
**23**	M	71	237	N/A	L	L	30	0	3	0	3	2	4	2	19
**24**	M	73	197	N/A	R	R	35	0	2	2	1	1	2	2	13
**Mean ± SD**	11F	72.0 ± 8.9	197.8 ± 50.1	-	2L	3L	40.3 ± 15.8	0.4 ± 0.7	2.6 ± 0.6	1.3 ± 0.9	2.3 ± 1.0	1.8 ± 1.0	4.5 ± 2.1	2.0 ± 0.9	16.2 ± 4.6
**Median**	-	73.0	198.5	-	-	-	39.5	0.0	2.5	1.0	2.0	2.0	4.0	2.0	14.5
**Range (low)**	-	51.0	120.0	-	-	-	5.0	0.0	2.0	0.0	0.0	0.0	2.0	1.0	10.0
**Range (high)**	-	85.0	300.0	-	-	-	76.0	2.0	3.5	3.0	4.0	3.0	8.0	4.0	29.0

SD represents standard deviation of population

^a^Medication doses represent total daily doses. Medications listed represent current, concomitant treatment at the time of incobotulinumtoxinA therapy.

### Selection and Administration of IncobotulinumtoxinA Treatments

**Fig 2:**
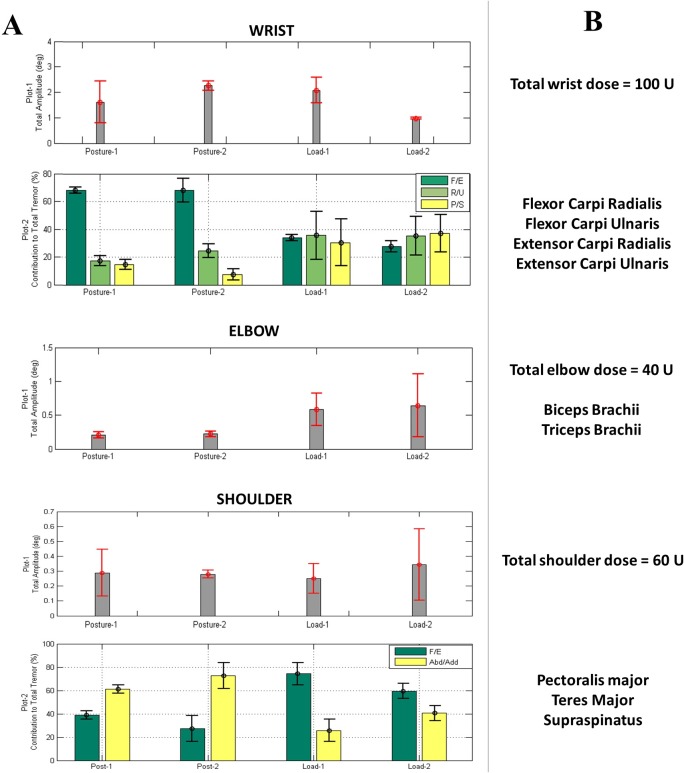
**Sample kinematic data showing (A) presence of tremor in the wrist, elbow and shoulder joints and (B) the ideal injection parameters determined using the kinematics with the injector’s best clinical judgement.** (A) Total tremor severity (plot 1) is displayed in angular RMS amplitude and the percent distribution of tremulous movement (plot 2) by 3 DOFs in the wrist and by 2 DOFs in the shoulder joint. Error bars indicate standard deviation over three trials. (B) Injector’s interpretation of the kinematic results showing selection of total dose allocated to wrist, elbow and shoulder muscle groups based on tremor severity and the muscles selected based on the amount of tremor present in each degree of freedom that each arm joint moves in.

Kinematics captured severity of tremor (angular RMS amplitude) and direction of the tremulous movement at each arm joint during each task. Fig 2A displays sample kinematic tremor measures showing quantification of tremor severity in wrist, elbow and shoulder joints (plots 1). For the wrist and shoulder joints, an additional plot calculated the distribution of the total tremor present in each degree of freedom that every joint moves in. Fig 2B demonstrates the injector’s interpretation of the kinematics showing that the selection of the total dose was based on total tremor severity and the muscles selected were based on the distribution of tremor at each arm joint during a task. In the example in Fig 2A, posture-2 task generated the most severe tremor in the wrist, and load-2 induced the largest tremor amplitude in elbow and shoulder joints. Muscle groups, which generate these fundamental movements, were then injected (Fig 2B). Thus, the kinematic measures for all participants and their individualized injection parameters ultimately developed a dosing table from the movement disorder’s clinical experience for each muscle and the dynamics of the movement at each joint.

For optimizing this therapy, a comparison in the change in tremor, measured kinematically, from pre-injection to six weeks (BoNT-A peak effect) and to sixteen weeks post-treatment was solely used to determine whether the BoNT-A dose or muscle sites needed to be altered. A reduction in total tremor at the six week follow-up indicated the appropriate muscles were targeted. An increase in BoNT-A dose was administered if the tremor could have been reduced further, as quantified by kinematics at post-injection assessments, and no side effects were perceived by participant (outlined in the Likert scale). A reduction in dose was indicated by the participant experiencing side effects, muscle weakness, as rated by the Likert scale for muscle weakness, a rating of 3+ or lower at the wrist flexion-extension and elbow flexion-extension using manual muscle testing, which indicates weakness in injected muscles lasting more than 4 weeks, and a significant difference in maximal grip strength when compared to baseline scores.

Participants (n = 24) were injected in their most bothersome arm. The mean total dose of incobotulinumtoxinA administered at the first treatment (week 0) was 169.0±62.9 U in 8.8±2.0 muscles (Table 2). The total dose for the second treatment was increased for 50.0% of participants (11/22), reduced for 13.6% of participants (3/22), and remained unchanged for 36.4% of participants (8/22). Between the second and third treatments, the total dose increased for 22.2% of participants (4/18), reduced for 11.1% of participants (2/18), and remained unchanged for 66.7% of participants (12/18). For the second treatment, the number of injected muscle sites increased to 10.1±2.0 muscles, which remained unchanged at the third treatment.

**Table 2:** Total injected dosage and number of muscles injected as determined by injector across all participants.

	Week 0 (First Injection)		Week 16 (Second Injection)		Week 32 (Third Injection)	
Patient ID	BoNT-A dose (U)	Num. of muscles injected	BoNT-A dose (U)	Num. of muscles injected	BoNT-A dose (U)	Num. of muscles injected
**1**	95	7	160	8	No injection^a^	
**2**	100	6	200	13	No injection^a^	
**3**	160	8	290	13	290	13
**4**	70	4	200	8	No injection^a^	
**5**	170	6	No injection^a^		No injection^a^	
**6**	300	9	300	9	300	9
**7**	200	11	100	11	100	11
**8**	200	9	150	9	150	9
**9**	195	9	300	12	300	7
**10**	185	10	185	10	200	13
**11**	100	8	200	11	200	11
**12**	200	8	185	9	185	9
**13**	170	10	170	10	165	10
**14**	200	11	260	11	300	11
**15**	100	9	No injection^b^		Withdrawn^c^	
**16**	200	10	200	10	260	13
**17**	300	11	300	14	Withdrawn^d^	
**18**	200	11	200	11	200	11
**19**	100	8	100	8	100	8
**20**	180	9	180	9	180	9
**21**	235	12	300	12	255	12
**22**	95	6	130	6	130	6
**23**	200	10	280	11	300	11
**24**	100	8	145	8	145	8
**Mean ± SD**	169.0 ± 62.9	8.8 ± 2.0	206.1 ± 65.8	10.1 ± 2.0	208.9 ± 71.0	10.1 ± 2.1

Dosing was in incobotulinumtoxinA units.

^a^ = Participants presented with minimal tremor at visit and injector made a clinical judgment against injection.

^b^ = Participants were not injected due to unattended study visit.

^c^ = Participant withdrew from study due to other health issues.

^d^ = Participant withdrew from study due to unwanted weakness.

The muscles selected and mean doses injected per muscle are listed in Table 3. The most frequently injected muscles during the first treatment were FCR and ECR (91.7%, 22/24). All participants were injected in the biceps for the second and third treatments.

**Table 3:** Mean injected dosage per arm muscle treated at each treatment time-point.

	Week 0 (First Injection)		Week 16 (Second Injection)		Week 32 (Third Injection)	
Muscles Injected	Mean ± SD	Num. of Patients (n = 24)	Mean ± SD	Num. of Patients (n = 22)	Mean ± SD	Num. of Patients (n = 18)
**Flexor carpi radialis (FCR)**	13.9 ± 4.9	22	14.5 ± 5.4	20	12.0 ± 5.6	15
**Flexor carpi ulnaris (FCU)**	12.4 ± 3.0	21	14.5 ± 5.8	20	12.3 ± 5.3	15
**Brachioradialis**	20.0 ± 0.0	2	27.5 ± 3.5	2	20.0 ± 0.0	1
**Extensor carpi radialis (ECR)**	15.7 ±5.4	22	16.5 ± 5.6	20	14.7 ± 6.7	15
**Extensor carpi ulnaris (ECU)**	16.2 ± 5.5	21	16.75 ± 5.9	20	15.7 ± 6.8	15
**Pronator teres (PT)**	15.3 ± 5.6	19	16.0 ± 5.8	21	15.3 ± 6.5	17
**Pronator quadratus (PQ)**	15.3 ± 5.6	19	16.0 ± 5.8	21	15.3 ± 6.5	17
**Supinator**	15.3 ± 5.7	17	18.2 ± 6.7	19	15.3 ± 6.7	16
**Biceps brachii**	28.6 ± 8.4	21	30.9 ± 8.5	22	30.3 ± 9.6	18
**Triceps**	28.7 ± 6.1	15	29.4 ± 7.8	18	30.6 ± 9.1	16
**Pectoralis major**	25.4 ± 6.3	13	25.7 ± 7.3	15	29.6 ± 10.7	13
**Teres major**	24.6 ± 7.5	12	25.0 ± 8.3	14	29.6 ± 11.4	12
**Deltoid**	28.0 ± 7.6	5	22.5 ± 5.2	6	30.0 ± 6.1	5
**Supraspinatus**	26.0 ± 9.6	5	21.7 ± 6.1	6	30.0 ± 8.4	6
**Infraspinatus**	0.0 ± 0.0	0	0.0	0	50 ± 0.0	1

All the dosages are in units of incobotulinumtoxinA. The mean values represent the average dose administered over the number of participants injected in the particular muscle. SD values represent standard deviation of population.

### Clinical and Kinematic Efficacy Results

Over the 38-week period comprising of three injection cycles, severity of action tremor (UPDRS item 21) was statistically significantly reduced [χ^2^(2) = 17.836,p<0.0005] from 2.6±0.5 at week 0 to 1.7±0.9 at week 16 (*p*<0.0005) and to 1.6±1.1 at week 32 (*p* = 0.001). Tremor severity in the untreated limb like rest tremor (UPDRS item 20) did not significantly change during study course.

**Fig 3:**
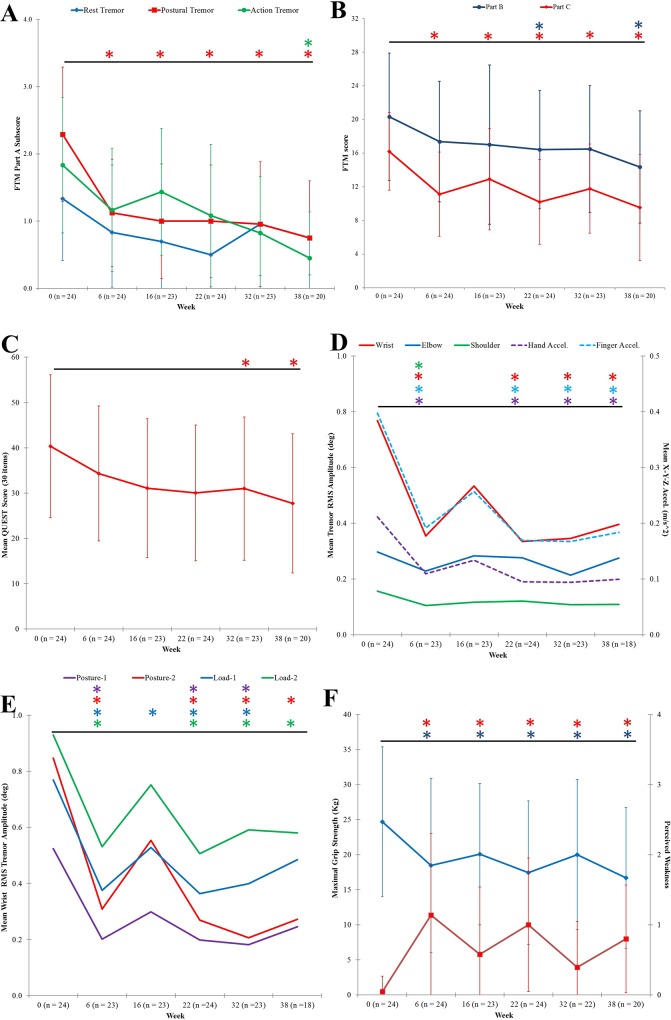
IncobotulinumtoxinA treatments significantly reduced severity of tremor and provided functional benefit for fine and gross motor tasks with mild muscle weakness in treated muscles. (A) Tremor severity, FTM part A sub-category score (max: /4 per task), significantly decreased. (B) Handwriting, spiral and line writing tasks showed significant improvement, signified by FTM part B summed score, and functional disability, FTM part C summed score (max: /4 per category, 8 categories in total), was significantly reduced. (C) Quality of life, measured by QUEST tallied 30-items (max: /4 per item), significantly increased. (D) Angular RMS tremor amplitude (primary y-axis) and hand and finger acceleration values (secondary y-axis) at each arm joint was averaged per time-point. Significant reductions in wrist and shoulder tremor amplitudes resembled change in hand and finger acceleration values. (E) Angular wrist tremor RMS amplitude for each scripted-task was significantly reduced. (F) Maximal grip strength (blue) was significantly reduced, but did not impair function, and perceived muscle weakness (red) yielded no significant change at injection visits. All plotted values are means for all participants per each time-point. Asterisks represented statistical significant change (p<0.05) compared to baseline. Error bars represent standard deviation of population.

Fig 3A illustrates the significant decline in FTM part A score assessing tremor severity during rest, posture, and action positions. Compared to week 0, means for FTM part A score for rest tremor did not meet normal distribution, thus Friedman’s test was utilized. Rest tremor was statistically significantly reduced during the BoNT-A treatment course, [χ^2^(5) = 13.809,p = 0.017]. Post hoc analysis revealed statistically significant reductions [χ^2^(5) = 37.568,p <0.0005] in postural tremor at baseline (median:2.0) to week 6 (median:1.0;p = 0.015), week 16 (median:1.0;p = 0.003), week 22 (median:1.0;p = 0.007), week 32 (median:1.0;p<0.0005) and to week 38 (median:1.0;p<0.0005). Action tremor, by post hoc analysis [χ^2^(5) = 21.348,p = 0.001], demonstrated significantly decreased changes in tremor from baseline (median:2.0) to week 38 (median:0.0;p = 0.002).

Total FTM part B sub-categorical scores rating the ability to write [F(5,95) = 2.286,p = 0.049,partial η^2^ = 0.107] and to pour liquids with both upper limbs was statistically significantly reduced [F(5,95) = 5.867,p <0.0005,partial η^2^ = 0.236] by a mean difference of 4.40±1.19 FTM points at week 22 (p = 0.23), 4.45±1.25 at week 32 (p = 0.31), and by 5.70±1.35 FTM points at week 38 (p = 0.007) when compared to week 0 (Fig 3B). Total FTM part C score, rating functional ability in eight categories, was significantly reduced [F(5,95) = 11.584,p <0.0005,partial η^2^ = 0.379] at all time-points with a final total FTM part C sub-score of 9.5 ±6.3 (*p*<0.0005), plotted in Fig 3B. Post hoc analysis revealed a decrease in functional disability caused by tremor by a mean difference in FTM part C sub-score of 5.25±0.943 at week 6 (p<0.0005), 3.95±0.82 at week 16 (p = 0.002), 5.65±0.92 at week 22 (p<0.0005), 4.25±0.96 at week 32 (p = 0.004), and 6.05±1.12 at week 38 (p<0.0005). The most disabling tasks at week 0 were drinking (2.8±0.8 FTM score) and working (2.5±1.0 FTM score). Across all participants, drinking ability was significantly improved by a mean difference of 0.95±0.29 at week 6 (p = 0.05) and by 0.95±0.21 at week 38 (p = 0.004). Working performance was statistically significantly improved at all time-points [F(5,90) = 4.751,p = 0.001,partial η^2^ = 0.209]. Other FTM categories such as eating, dressing and hygienic activities significantly improved and functional disability due to tremor did not return to baseline severity. ET participants reported elevated quality of life, measured by QUEST (Fig 3C). Mean total QUEST score was significantly reduced [F(5,95) = 4.620,p = 0.001,partial η^2^ = 0.196]; post hoc analysis showed quality of life significantly improved at the time of and following the third treatment, by a mean difference of 9.45±2.69 at week 32 (p = 0.035) and by 10.50±2.91 at week 38 (p = 0.028), when compared to baseline.

Kinematic tremor assessments allowed objective monitoring of tremor severity before and after incobotulinumtoxinA therapy. Fig 3D displays angular tremor RMS amplitude and acceleration captured at the finger and hand values analyzed together over two postural (”posture-1” and “posture-2”) and two weight-bearing (”load-1” and “load-2”) tasks. Though joint angles and acceleration, which is quantified by the sum of acceleration in the X-, Y- and Z- axis, indicate different characteristics, they both represent tremor severity. Mean wrist RMS amplitude was significantly reduced [F(2.297,85) = 7.594,p = 0.001,partial η^2^ = 0.309] by mean difference of 0.39±0.10 at week 6 (p = 0.014), by 0.43±0.12 at week 22 (p = 0.027) and by 0.41±0.09 at week 32 (p = 0.005). Mean elbow tremor amplitude during both weight-bearing tasks (load-1: χ^2^(5) = 13.587, p = 0.018; load-2: χ^2^(5) = 11.714, p = 0.039] produced statistically significantly different changes over treatment course. Mean elbow tremor during posture-2 (arms outstretched with palms facing inwards) [χ^2^(5) = 14.413,p = 0.013] demonstrated a significant decrease in tremor by a mean difference of 2.278±0.62 at week 32 (p = 0.004). Hand tremor acceleration significantly decreased [χ^2^(5) = 27.937,p<0.0005] across all time-points except at week 16, correlating to the change in wrist tremor amplitude (Fig 3D). Weight-bearing tasks produced the largest acceleration at the finger and hand.

Analyzing tremor per task (Fig 3E), load-2 produced the largest mean tremor amplitude of 0.9±0.7 RMS degrees (median: 0.81) in the wrist at week 0 which was significantly reduced [χ^2^(5) = 20.667,p = 0.001] to 0.5±0.6 degrees at week 6 (median:0.34;p<0.0005). Similar reduction in wrist tremor was observed during posture-1 [χ^2^(5) = 18.921,p = 0.002], posture-2 [χ^2^(5) = 22.636,p <0.0005], and load-1 [χ^2^(5) = 22.635,p <0.0005] tasks for all time-points excluding week 38 for posture-1 and for load-1.

Significant change in maximal grip strength [F(2.730, 49.132) = 11.155,p <0.0005,partial η^2^ = 0.383] coincided with peak effect of toxin but did not affect arm functionality or quality of life (Fig 3F). Maximal grip strength was significantly reduced from 24.7±10.7 kg at week 0 to 18.5±12.4 kg at week 6 (mean difference of 6.51±1.54; *p* = 0.007). A significant reduction in maximal grip strength however did not indicate any impact on arm function, demonstrated on a Likert scale for self-reported perceived muscle weakness (Fig 3F, red line). At week 6, 12 participants (50%) reported a Likert score of 0, no weakness, two participants (8.3%) reported a 1, mild weakness with no loss in function, seven participants (29.1%) reported a 2, moderate weakness in injected muscles, and three participants (12.5%) reported a 3 indicating marked arm weakness. Following third treatment at week 38, eight participants (40%) experienced no weakness; eight and four participants reported a score of 1 and 2, respectively.

## Discussion

This is the first study that uses whole limb kinematics to segment complex movements at multiple joints comprising of tremor in order to determine if efficacy and safety of incobotulinumtoxinA (BoNT-A) as a focal therapy is achievable. Kinematics provides an objective readout of the angular motion of tremor acting at each joint during a variety of tasks thereby providing the composition of tremor. This composition is unique for every patient and thus the selection of contributing joints, muscles that move the joint in the affected degrees of freedom and the dosing of these muscles can be individualized. An objective, repeatable platform of measuring the biomechanical properties of the tremor means that the same measurements can be carried out at any time point after the injection to determine the effect of injection. Such tools can record motion at multiple joints simultaneously, for an extended period of time, that can be averaged and thereby give a comprehensive dynamic view of the tremor. Visual assessment does not meet any of these criteria.

In the process of injection determination, the injector initially chooses a total dose for injection permitting dose allocation to appropriate muscles generating the tremulous movements. Such targeting immediately individualizes the injections to the kinematic signature of the patient. Subsequent optimization by measuring the effect of the injection at subsequent visits is also possible, as demonstrated in this study. This longitudinal study demonstrates for the first time sustained functional improvement in ET by effective use of kinematics in personalizing incobotulinumtoxinA injections with a low incidence of weakness. By using such technology, a standardized method to assess tremor has been established and these results can be used to improve focal therapy thereby paving a way to offer clinicians and patients with alternate options for treating tremor.

ET patients who seek treatment suffer from functionally disabling tremor which restricts performance of every-day activities [1]. As 30% of patients do not respond to standard pharmacological medication and yet another 30% who start drug therapy will discontinue treatment due to side effects, an effective tremor therapy is needed [4,6]. Thalamotomy and thalamic stimulation is often age restricted, has strict guidelines including cognitive status, and is not accessible to many due to the requirements for a specialized centre. In addition, significant irreversible complications including dysarthria and gait difficulties can occur. As such surgical therapies are often restricted to a small group of severally disabled patients, which highlights the need for a targeted treatment such as BoNT-A injections. Prior studies have utilized BoNT–A injections as focal treatment, though significant finger and wrist muscle weakness has curtailed its use, despite its promising clinical benefit [10,11,18–20]. To improve BoNT–A efficacy and to reduce incidence of unwanted effects, this study addressed several major prior study limitations. These limitations include inability to determine the joints and their dynamics for the involved tremor, fixed and/or randomized dosing, subjective and/or fixed number of muscles selected, lack of individualization of injections to the participant’s tremor, and number of injection cycles.

Accurate measurement of movement in multiple degrees of freedom at each upper limb joint is the first unmet need that has been addressed by our technology. Selection of tremulous muscles has previously been established by using a fixed method, injecting only flexor and extensor wrist muscles [10,18,19], by using single joint surface EMG electrodes [8], or by combining accelerometry and surface EMG [11]. The assumption here is that the tremor at the wrist is mainly unidirectional (F/E) and contributions from the elbow and shoulder joints are not measurable. Hence, accurate localization or segmentation of tremor at wrist, elbow and shoulder joints was not performed. This creates significant segmentation errors as a significant portion of the tremor originates from movements other than wrist F/E and indeed from proximal joints. Unlike accelerometers which provide tremor amplitude data for the entire arm, the sensors employed in this study allowed independent characterization of motion at the wrist, elbow, and shoulder joints, which is a difficult task by visual assessment or accelerometric and surface EMG measurements alone [13,15,16]. Based upon the composition of movement dynamics at multiple joints and in multiple directions at each joint, the contributing muscles were selected (Fig 2A). Injection patterns are thus tailored to each participant’s kinematics (Fig 2B) instead of using visual methods or by standard set of injections, utilized in prior studies.

Fixed and randomized dosing, preselected muscles that may not actually be involved, while allowing a standardized approach to injection, fails to take into account an important aspect of significant individual variation in tremor. Applying objective kinematic technology to every patient uniquely provides a “read-out” of the patient’s own tremor. This approach can thus reduce potential unwarranted weakness and indeed improve efficacy as the correct joints and muscles are targeted. Dose-dependent limb weakness limited functional efficacy of BoNT–A as shown in several earlier studies that utilized a fixed- or randomized-dosing method [10,18–20]. In addition, Brin MF. *et al* did display reduced postural tremor severity by using accelerometers, but could not show functional benefit following a BoNT–A treatment [10]. Hence, in this study, individualizing injection patterns optimized tremor therapy by characterizing the joints involved and by quantifying the angular displacement of tremor in each degree of freedom, an analysis only capable by the use of kinematics. Based upon the clinician’s judgment, muscles predominantly contributing to the total tremor were selected, though these muscles may vary somewhat depending upon the personal choices of the clinician.

An important unmet need with clinical assessments is the ability to change the dosing at a subsequent visit. Since visual assessment does not provide an objective record of the patient’s prior limb motion, there is no objective way to compare the limb motion at subsequent visits. Kinematics are quantitative and repeatable, thereby providing a simple way for the clinician to determine the pattern of the original joint involvement and then continue optimization at any visit that is desired after that. In this study, we were able to achieve this optimization. Changes in BoNT-A dosages between treatments were calculated to optimize response by comparing the severity of tremor pre- and post-injection solely using kinematics, a personalized, targeted therapy unachievable by oral medications.

By using kinematic methodology, significant functional benefit, particularly for eating, drinking and working performance, was achieved six weeks following the first treatment and throughout the study course along with reductions in tremor severity during rest, postural, and action tasks (Fig 3A–3E). These benefits generated a significant improvement in quality of life scores, ranging across physical, psychosocial, communication, hobbies/leisure, and work/finance activities, at week 32 and 38 (Fig 3C), which has not been achieved in any of the prior BoNT-A studies for upper limb essential tremor [1]. Along with these physical and functional benefits, maximal grip strength was statistically significantly reduced at study visits, but functional strength was only minimally affected as demonstrated by the Likert perceived weakness scale (Fig 3F). A mild decrease in maximal grip strength was attributed to a modest toxin effect to the neighboring finger flexors by transfascial diffusion and/or to other synergistic muscles. Finger muscles were not directly treated because the kinematic tremor analysis did not include finger sensors. Thus, benefits of using kinematically-guided injections achieved relief of tremor, functional benefit and demonstrated significantly less muscle weakness compared to prior studies. These results indicate that incobotulinumtoxinA injection parameters determined using kinematics can effectively and safely reduce upper limb tremor, while keeping weakness related side-effects low. A clinician familiar with the anatomy and who is knowledgeable about BoNT-A dosages typically given to these muscles for other indications, such as spasticity, now has the ability to confidently treat tremor using kinematics as guidance. By accurately pinpointing joint dynamics in ET, individualization and optimization of tremor treatment is now possible.

Non-blinded injections and no treatment comparator were limitations of this study. However, in this longitudinal study, outcomes were kinematically and objectively determined with serial injections and hence a persistent placebo response is unlikely. Blinded studies with BoNT–A are difficult as weakness is obvious and easily perceived by participants and investigators. Similarly, cross-over designs are challenging as it is impossible to determine a true return to baseline in injected participants for accurate cross-over time. Final muscle injection pattern was determined by the treating physician and may vary. However, this allows even better individualization and flexibility. The study did not compare visually guided versus kinematically guided injections as the lack of tolerability and efficacy with injections based on visual assessments has already been demonstrated in the literature. Sample size was similar to previous literature [10,19]. As tremor is variable throughout a given day and participants were assessed while on their anti-tremor medications, severity fluctuations could have introduced error during each visit. Thus, participants were assessed around the same time of day.

It is also important to note that only one of the arms was injected to allow participants’ functionality of at least one limb in case of unwarranted side effects of weakness. Although the most affected arm for functionality was treated, it is possible that even further improvements in quality of life can be achieved if both arms had been injected from the start.

This study clearly demonstrates that utilizing an objective, kinematically-based assessment of upper limb tremor provides a clinician with critical guidance for selecting which joints are affected and in what proportion. This allows for targeted, individualized muscle selection to significantly improve efficacy of consecutive incobotulinumtoxinA injections for tremor. For the first time, incobotulinumtoxinA injections have effectively treated essential tremor and enhanced the quality of life of patients suffering with essential tremor by improving functional ability of their whole arm.

## Supporting Information

S1 Fig Images depicting standard scripted-tasks performed by each participant during kinematic recording sessions. (A) Postural position (posture-1) with shoulders flexed at 90° with arms extended anteriorly and pronated (palms facing downwards). (B) Postural position (posture-2) position with shoulders flexed at 90° with arms extended anteriorly, palms facing inwards. (C) Functional task (load-1) with the participant holding an empty cup in front of body with elbow and proximal arm unsupported (D) Functional task (load-2) holding a cup with a one-pound weight in front of body with elbow and proximal arm unsupported.
(TIFF)

S2 Fig Images depicting sensor placement. Placement of Biometric® motion sensors along arm: shoulder electrogoniometer, elbow electrogoniometer, wrist electrogoniometer, accelerometers placed on forearm, hand and third finger.
(TIFF)

S1 File Comprehensive kinematic assessment protocol. (DOCX)

S2 File Transparent reporting of evaluations with nonrandomized designs (TREND) checklist of the study design, outcomes, and interpretation. (PDF)

S3 File The full study protocol approved by the Western University Health Sciences Research Ethics Board. (DOC)
