# Supplementary figures and images for: Functional Ability Improved in Essential Tremor by IncobotulinumtoxinA Injections Using Kinematically Determined Biomechanical Patterns – A New Future
Source: PLoS One. 2016 Apr 21;11(4):e0153739. doi: 10.1371/journal.pone.0153739 (PMC4839603; doi:10.1371/journal.pone.0153739)

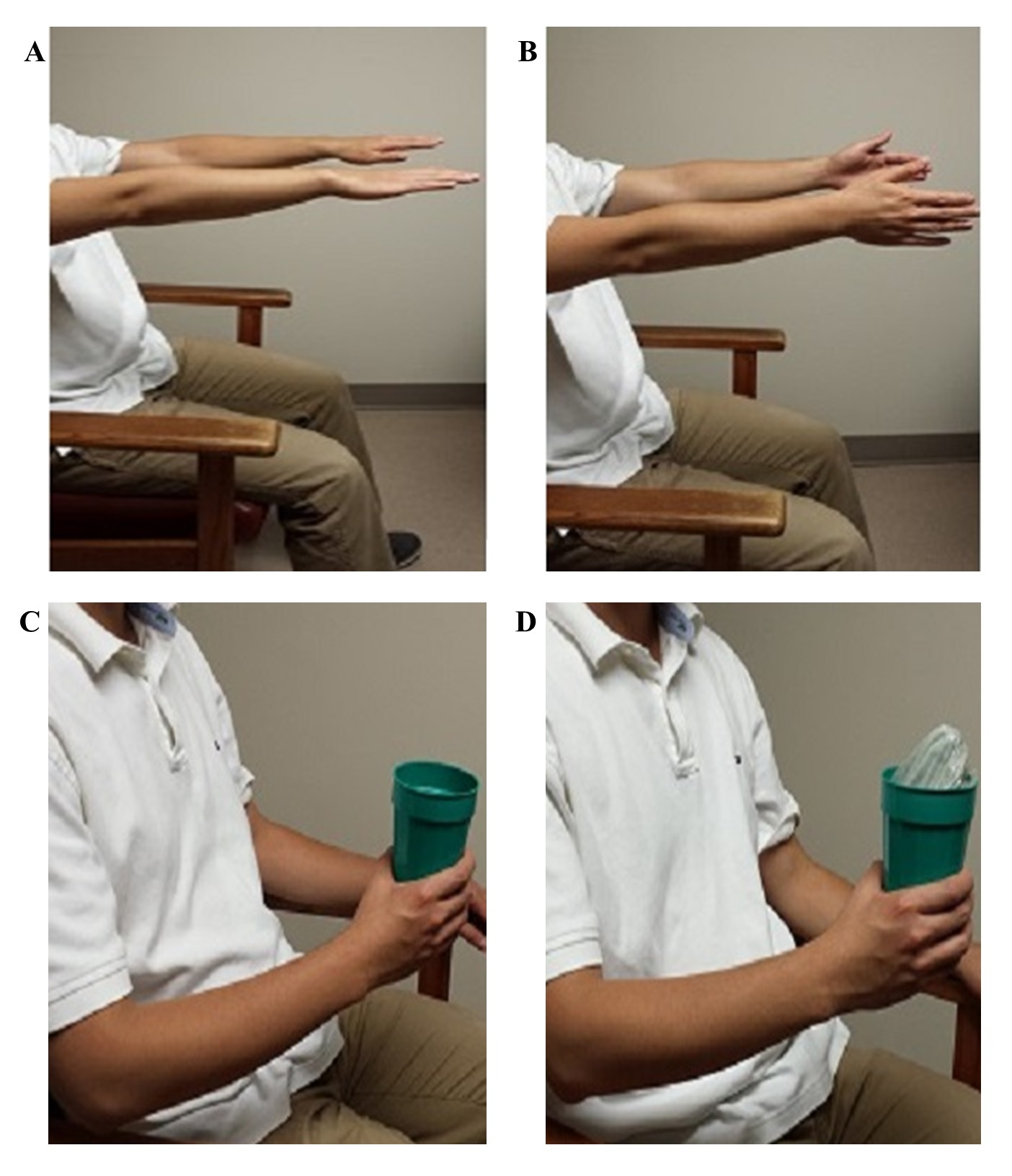

Supplement: S1 Fig — (A) Postural position (posture-1) with shoulders flexed at 90° with arms extended anteriorly and pronated (palms facing downwards). (B) Postural position (posture-2) position with shoulders flexed at 90° with arms extended anteriorly, palms facing inwards. (C) Functional task (load-1) with the participant holding an empty cup in front of body with elbow and proximal arm unsupported (D) Functional task (load-2) holding a cup with a one-pound weight in front of body with elbow and proximal arm unsupported. (TIFF) [file pone.0153739.s001.tiff]

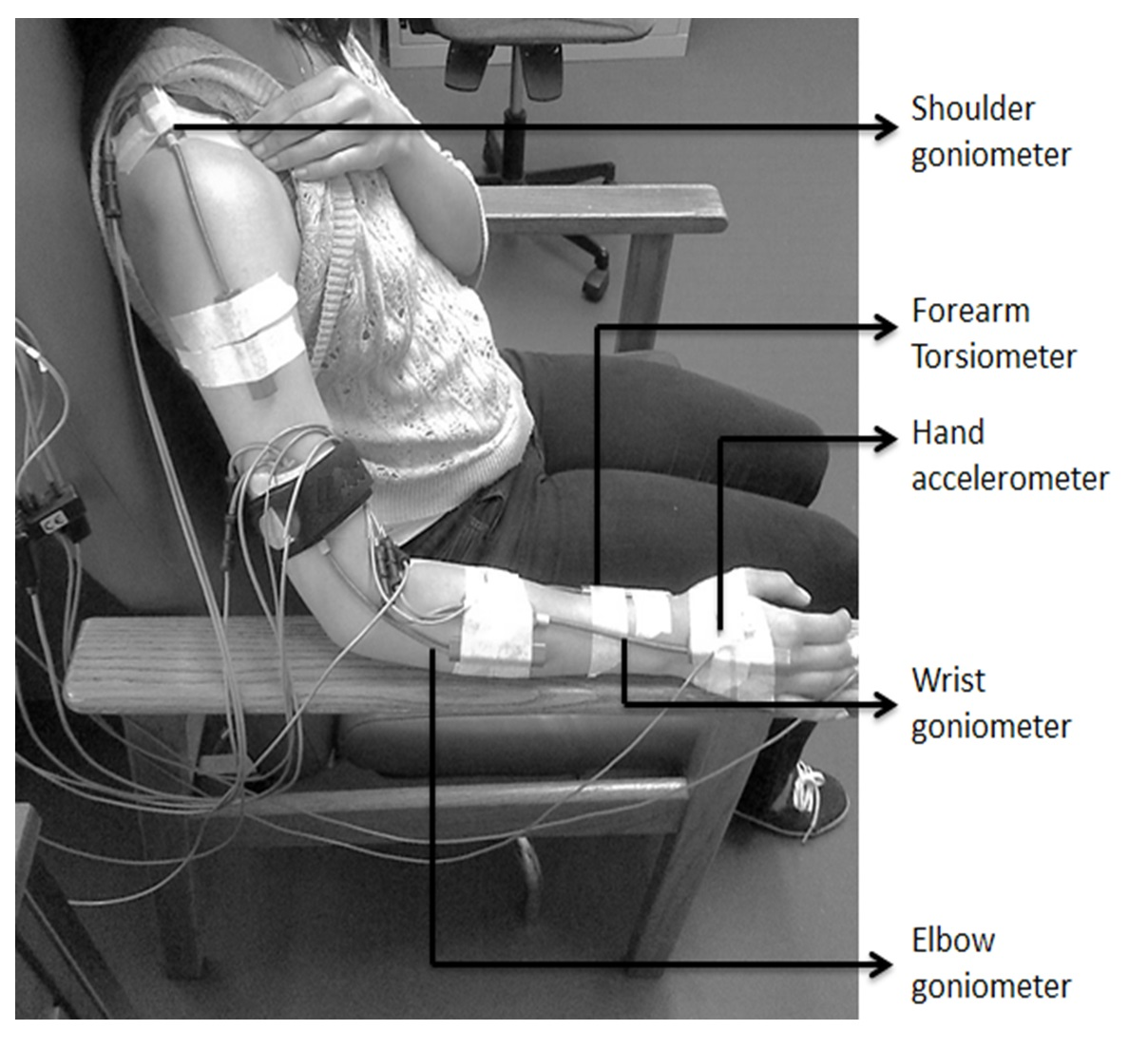

Supplement: S2 Fig — Placement of Biometric® motion sensors along arm: shoulder electrogoniometer, elbow electrogoniometer, wrist electrogoniometer, accelerometers placed on forearm, hand and third finger. (TIFF) [file pone.0153739.s002.tiff]
