# Supplementary material for: Functional Ability Improved in Essential Tremor by IncobotulinumtoxinA Injections Using Kinematically Determined Biomechanical Patterns – A New Future
Source: PLoS One. 2016 Apr 21;11(4):e0153739. doi: 10.1371/journal.pone.0153739 (PMC4839603; doi:10.1371/journal.pone.0153739)
Supplement: S1 File — (DOCX) [file pone.0153739.s003.docx]

**Supporting Information:** Comprehensive kinematic assessment protocol

**Title:** Functional ability improved in Essential Tremor by incobotulinumtoxinA injections using kinematically determined biomechanical patterns – A new future

**Authors:** Olivia Samotus,^1,2^ Fariborz Rahimi,^1,3^ Jack Lee,^1^ Mandar Jog^1,2*^

**Keywords:** Essential tremor; functional disability; movement disorders; Botulinum toxin type A; kinematics

______________________________________

^1^Department of Clinical Neurological Sciences, London Health Sciences Centre – Lawson Health Research Institute, London, Ontario, Canada

^2^Schulich School of Medicine and Dentistry, Western University, London, Ontario, Canada

^3^Department of Electrical and Computer Engineering, University of Bonab, Bonab, East Azerbaijan, Iran

***Corresponding Author:**

Email: mandar.jog@lhsc.on.ca (MJ)

**Email addresses of authors:**

Olivia Samotus: osamotus@uwo.ca

Fariborz Rahimi: frahimi@bonabu.ac.ir

Jack Lee: jlee544@alumni.uwo.ca

Mandar Jog: mandar.jog@lhsc.on.ca

______________________________________

**Supporting Information**

**Kinematic Experimental Tasks**

Sensor calibration was completed with the forearm supported and with the hand fixed against a vertical plane in neutral F/E, R/U, and P/S positions, and was held for five seconds. Additional sensor calibration was performed with the participant’s arm held straight while standing, elbow extended with fingers pointing down for five seconds. A total of four consecutive scripted tasks were repeated three times in series (figure e-1A–D). 20 seconds were allotted to each task which involved two postural positions (focusing on F/E and R/U movements), and two weight-bearing functioanl tasks.

[Fig A]

**Kinematic Assessment Set-up**

Figure e-2 displays the placement of a total of four motion recording sensors attached over the wrist, elbow, and shoulder arm joints. Electrogoniometers measured wristt tremor, by angular position, in two degrees of freedom, flexion/extension (F/E) and radial/ulnar deviation (R/U) (SG150, Biometrics Ltd). Wrist tremor in the pronation/supination (P/S) plane was measured by a torsiometer placed on the dorsal surface of the forearm (Q150, Biometrics Ltd.). Thus, the torsiometer provided the third angular degree of freedom of rotational motion about the wrist. Elbow tremor was captured an electrogoniometer in one degree of freedom, flexion/extension (F/E). An electrogoniometer placed on the shoulder measured two degrees of freedom, flexion/extension (F/E) and abduction/adduction (Abd/Add). Sensors were attached using 3M hypoallergenic micropore medical grade tape (Ref#: 1530–1).

Motion sensor data was collected at 1500Hz by TeleMyo™ 2400T G2 and PC interface (MyoResearch XP Master Edition 1.08.09, Noraxon®). Recordings at each joint were mutually exclusive with each sensor recorded data only from a particular joint. Accelerometer sensor data was additionally collected as a comparison tool to prior studies and was not used for determining tremor composition.

[Fig B]
