# Supplementary material for: Functional Ability Improved in Essential Tremor by IncobotulinumtoxinA Injections Using Kinematically Determined Biomechanical Patterns – A New Future
Source: PLoS One. 2016 Apr 21;11(4):e0153739. doi: 10.1371/journal.pone.0153739 (PMC4839603; doi:10.1371/journal.pone.0153739)
Supplement: S3 File — (DOC) [file pone.0153739.s005.doc]

**1. PROTOCOL SUMMARY**

**Trial Title and Protocol Number/Code**

*Trial Title:* Use of kinematic assessment of hand tremor pre- and post-treatment with botulinum toxin type A in essential tremor and Parkinson disease.

*Protocol Number:* 18445

**Background and Rationale**

*Botulinum Toxin A (BoNT A) and Essential Tremor (ET)*

Almost four decades since its introduction, BoNT A has become widely used in numerous medical and therapeutic applications, particularly for the treatment of hyperkinetic movement disorders such as tremor, tics, myoclonus, and spasticity [1, 2]. Tremor is one of the most common movement disorders; ET is a leading cause of neurology referral for clinical assessment of tremor [3]. Despite early doubts [4] and reported lack of benefit in tremor reduction [5], efficacy of BoNT A has been demonstrated for tremor of the hand [6,7], head [8,9], and even voice [10,11].

*BoNT A and Idiopathic PD (IPD)*

Behind ET, IPD is the second most common cause of tremor. BoNT A therapy has been investigated for treatment of tremor in this population as well as in ET. While tremor is a benign symptom of IPD, it is frequently targeted for treatment as it often leads to social anxiety and inconvenience in daily living. Therefore, tremor has become an important indicator in clinical standard of care for IPD severity, progression, and medication inadequacy. The majority of studies reporting use of BoNT A therapy have studied ET patients [6,7,12], though a few studies have used IPD patients [13,14,15] Therefore, there is a deficit in understanding of the effects of BoNT A therapy for IPD tremor, and the complete lack of literature regarding BoNT A therapy efficacy for different types of tremor. The present study aims to fill this knowledge gap by investigating the differential effect of Xeomin® on ET compared to IPD.

*Advantages of BoNT A*

To date, efficacy in treatment of essential tremor by oral medication has been sup-optimal for all individuals. Propranolol and primidone are the most commonly prescribed medications, yet they have only limited efficacy for tremors of high amplitude, and often cause unpleasant side effects such as hypotension, as well as mood, behavioural, and cognitive impairment. In IPD, tremor is one of the least responsive symptoms to L-dopa therapy. Therefore, effective BoNT A therapy for treatment of tremor may be more effective at reducing tremor than the currently prescribed oral medications for either ET or IPD, and for IPD patients, could prevent unnecessary increases in L-dopa dosage. Management of tremor via local intramuscular injection of BoNT A introduces an optimal alternative to increasing medication prescription and dosage in ET [6,7,15] and IPD [15]. Collectively, these findings suggest that there is a substantial benefit in studying the efficacy of Xeomin® in tremor management in ET and IPD.

*Kinematics*

While most studies of tremor treatment with BoNT A have used clinical rating scales to assess upper limb tremor, some have also used accelerometry as an objective kinematic measure of tremor severity [6,8,9,16] Accelerometry, however, is limited not only in its ability to capture and characterize tremor, but also in its ability to determine injection efficacy. Accelerometry measures the overall amplitude and frequency of tremor, but does not capture the contribution of individual muscle groups, is unable to measure the contribution of different joints (wrist, elbow, shoulder) to the tremor, and cannot be used to separate movement in different degrees of freedom (e.g. flexion/extension, radial/ulnar deviation and pronation/supination for the wrist). In the proposed study, we expand on previous studies by including a greater scope of kinematic measurements (using goniometers, inclinometers, and accelerometers) to increase our understanding of tremor and efficacy in the use of Xeomin® in tremor management.

*Kinematic Assessment vs. Clinical Assessment*

Preliminary work on kinematic recordings of tremor in ET and IPD indicates that upper limb tremor is comprised of a combination of complex movements and often involves components from different muscle groups at different joints (flexor/extensor, ulnar/radial deviators, and pronator/supinator for the wrist; flexors/extensors for the elbow and flexors/extensors and abductors/adductors for the shoulder). This finding supports the practice of injection individualization [6]. The biomechanics of upper limb tremor are multi-faceted and complicated; injection practice should reflect this complexity. It is likely not reflected by current gold-standard clinical assessments that rely on visual inspection alone, and may explain the inability of previous studies to demonstrate efficacy of BoNT A injection therapy for the treatment of tremor [5]. Improving injection accuracy by pinpointing sites of tremor origin and calculating appropriate dosage may be the key to achieving efficacy with BoNT A therapy. Kinematic biomechanical assessment tools may help to quantify, understand, and improve efficacy of injection practices. Presently, the objective decomposition of tremor into all components and the determination of injection parameters based on this data has not been adequately researched in the literature [17]. The present project attempts to fill this knowledge gap, allowing for the development of accurate and reliable kinematic recording methods, biomechanical analysis methods and consequently, development clinical expertise in tremor management using BoNT A injections.

*References:*

1. Schantze. J. and Johnson E. A., Properties and Use of Botulinum Toxin and Other Microbial Neurotoxins in Medicine. Microbiological Reviews 56(1), 1992.
2. Gerald V., Evidente H. and Adler C. H., An Update on the Neurologic Applications of Botulinum Toxins, Curr Neurol Neurosci Rep (2010)
3. Benito Leon, J. & Louis, E.D. (2006). Essential tremor: emerging views of a common disorder. Nat Clin Pract Neurol, 2, 666 78.
4. Pullman S.L., Elibol B., Fahn S., Modulation of parkinsonian tremor by radial nerve palsy, Neurology. 1994 Oct;44(10):1861-4.
5. Gironell A.and Kulisevsky J., Diagnosis and management of essential tremor and dystonic tremor, Ther Adv Neurol Disord (2009) 2(4) 215–222
6. Jankovic J, Schwartz K, Clemence W, Aswad A, Mordaunt J. A randomized, double-blind, placebo-controlled study to evaluate botulinum toxin type A in essential hand tremor. Mov Disord. 1996 May; 11(3):250-6
7. Brin, M. F., Lyons, K. E., Doucette, J., et al. (2001). A randomized, double masked, controlled trial of botulinum toxin type A in essential hand tremor. Neurology, 56, 1523- 8.
8. Pahwa R, Busenbark K, Swanson-Hyland EF, et al. Botulinum toxin treatment of essential head tremor. Neurology 1995;45:822–824.
9. Wissel J, Masuhr F, Schelosky L. Quantitative assessment of Botulinum toxin treatment in 43 patients with head tremor. Mov Disord 1997;12:722–725.
10. Warrick P, Dromey C, Irish JC, et al. Botulinum toxin for essential tremor of the voice with multiple anatomical sites of tremor: a crossover design study of unilateral versus bilateral injection. Laryngoscope 2000; 110:1366–1374.
11. Hertegard S, Granqvist S, Lindestad PA. Botulinum toxin injections for essential voice tremor. Ann Otol Rhinol Larnygol 2000;109:204–209.
12. Adler C.H., Bansberg S.F., Hentz J.G., et al.: Botulinum toxin type A for treating voice tremor. Arch Neurol 2004, 61:1416–1420.
13. Jankovic J. & Schwartz K., Botulinum toxin treatment of tremors. Neurology, 1991 41, 1185-8.
14. Trosch R. M. and Pullman S. L., Botulinum toxin a injections for the treatment of hand tremors, Movement Disorders 9(6), 601–609, 1994
15. Sheffield J.K. and Jankovic J., “Botulinum toxin in tic disorders and essential hand and head tremor, ” in Manual of Botulinum Toxin Therapy, D. D. Daniel Truong and M. Hallett, Eds. Cambridge University Press, 2009.
16. Henderson J.M., Ghika J.A., Van Melle G., Haller E. and Einstein R., Botulinum toxin A in non-dystonic tremors. Eur Neurol. 1996;36(1):29-35.
17. Rozman J., Bartolic A. and Ribaric S., A new method for selective measurement of joint movement in hand tremor in Parkinson’s disease patients. J Med Eng & Tec, 31(4), 2007

**Trial Objectives**

1. Investigate the efficacy of Xeomin® injections based on kinematic parameters in the treatment of upper limb tremor in Parkinson disease (IPD) and essential tremor (ET)
2. Assess the benefit of using kinematic measurement tools for determining BoNT A injection parameters
3. Study upper limb tremor composition in ET and PD patients using kinematic assessment

*Research questions:*

1. Is there a significant improvement in tremor severity, as measured by standardized clinical assessment tools, post Xeomin® injection using kinematic guided injection parameters, for both IPD and ET patients?
2. Is there a significant reduction in overall tremor amplitude (as measured kinematically) post Xeomin® injection using kinematic guided injection parameters for both IPD and ET?
3. What is the difference in composition of tremor at baseline between two subgroups of participants: those with > 8 points change on the standard Fahn-Tolosa-Marin Tremor Rating Scale (FTM scale) and those with ≤ 8 points change the FTM scale?

**Study Design and Duration**

*Study Design:* open-label, pre-/post-treatment study design.

A pilot study design was selected to investigate the effect of this particular BoNT A formulation on both IPD and ET when kinematic data is used to determine injection parameters. A control group is difficult for this study given that it would not be appropriate to inject individuals without tremor. Additionally, at this stage of the study, with very little published literature on this BoNT-A formulation, completing a randomized study between two BoNT A formulations would not be reasonable. Instead, we have chosen to expose all participants to the same treatment.

Seventy participants will complete six injection cycles over 86 weeks. Participants will be allocated to one of two groups IPD (35 participants) or ET (35 participants) following assessment by a Movement Disorders Neurologist (MDN). To optimize BoNT A therapy efficacy, adjustments to injection site and units injected will be made as required later in the study based on benefit observed from previous injection cycles. Clinically judged improvement (by the MDN) and patient reported improvement will be the determinants of need for dose/injection site modification. If the first injection was sufficient in reducing tremor to an acceptable level, the same injection will be repeated. If not, then the injection parameters will be modified by the MDN. Routine clinical use of BoNT-A for tremor usually requires two consecutive injection cycles to optimize effect.

IPD participants will complete all visits in their optimal “on” state relative to their PD medications. Participants will be asked to take other medications as normal. They will be instructed not to ingest caffeine on the day of assessment to minimize confounding of tremor data. For all participants, the tremor dominant hand will be targeted for injection.

Xeomin® will be re-constituted by the same clinical nurse for all injections. This is done to ensure methodological consistency. To reduce bias, a single MDN will complete all injections for both groups. The MDN will determine parameters of injection using kinematic data for each injection course. The injecting MDN will be blinded to the kinematic/video recordings, but will make decisions from the analyzed data. Needle EMG may be utilized at the discretion of the MDN to ensure accuracy of injection sites in accordance with routine clinical practice. The injecting MDN will receive training in interpreting of kinematic data results prior to the initiation of the study.

*Duration*: Participants will receive a total of 6 injections and attend 12 study sessions over the course of 86 weeks. Duration of each participant visit will be approximately 75-90 minutes.

**Total Number of Sites and Number of Canadian Sites**

*Total Number of Sites:* 1

*Number of Canadian Sites:* 1 (London Health Sciences Centre University Hospital, London, Ontario, Canada)

**List of Investigators**

*LHSC University Hospital:* Dr. Mandar Jog

**Sample Size**

*Number of subjects*: 70

Sample size justification:

| Alpha error | Alpha error = .05, two-sided. |
| --- | --- |
| Statistical power | 1-β = 0.95 |
| Difference detectable with specified sample size | Effect size = .44 |

*Sample size calculations:* Power calculations are based on our previous pilot study as well as a review of relevant literature. We have based all power calculations at 1-ß = .95 to ensure efficacy for the primary endpoint. Power calculations were conservatively based on *F*-test paradigm for ANCOVA using G*Power 3.1.2 software package. Alpha error = .05.

There were no relevant studies on Xeomin® and hand tremor found in the literature search. To calculate effect size for efficacy of hand tremor management using Botulinum toxin A, we have used a randomized, double masked study of efficacy in essential tremor by Brin, et al. (2001). They calculated the effect size for N = 133 (allocated to two groups high (100 units) and low (50 units)). For the current power calculation the low unit group was used as a conservative estimate. For tremor scale measures (primary endpoint) we used an effect size = .44 (Brin, et al. 2001). Based on previous studies in our group, we would anticipate a lower effect size for the kinematic outcome measures (secondary endpoint) estimated at .33 (overall tremor amplitude).

The target sample size for this study is N = 70 (35 per group for both IPD and ET), which will allow for 1- ß = .95 for an effect size for the primary endpoint of .44 and critical *F* = 3.98. This sample size will allow for an attrition rate of 20% while maintaining a 1-ß = .90 (*F* = 4.02) and an attrition rate of 37% while maintaining 1-ß = .80 (*F* = 4.08). Given the length of the study (5 visits over 8 months) we estimate a 30% attrition rate and have planned conservatively for that.

**Patient Population**

*IPD:* botulinum toxin naïve consenting males and females, aged 18-80 years, diagnosed with stage H&Y 2-3 idiopathic Parkinson disease by the UK Brain Bank Criteria, with tremor as their primary and most bothersome symptoms, who have experienced a stable parkinsonian medication regime for at least 6 months prior to study enrolment.

*ET:* botulinum toxin naïve consenting males and females, aged 18-80 years, diagnosed with essential tremor by a qualified neurologist, presenting with tremor in their motor dominant hand, who have experienced a stable tremor medication regime for at least 6 months prior to study enrolment.

**Inclusion Criteria**

1. Consenting male and female participants aged 18 years to 80 years
2. IPD: individuals diagnosed by UK Brain Bank Criteria with stage H&Y 2-3 idiopathic Parkinson disease (eligible only if tremor is the patient’s primary and most bothersome symptom as determined by clinical exam and patient report)
3. ET: individuals diagnosed with ET presenting with hand tremor in their motor dominant hand
4. Stable IPD/ET medication management for 6 months prior to study enrolment
5. Individuals who are botulinum toxin naïve

**Exclusion Criteria**

1. History of stroke
2. History of ALS or myasthenia gravis
3. Muscle weakness or any related compartmental muscle syndrome
4. Smoking
5. Offending medications (lithium, valproate, steroids, amiodarone, or beta-adrenergic agonists such as salbutamol)
6. Individuals prescribed zonisamide
7. History of allergic reaction to botulinum toxin or history of severe side effects in response to botulinum toxin injections
8. Contraindications per the Xeomin® drug monograph
9. Pregnant women

**Drug Formulation**

*Drug formulation:* Single-use, sterile 100 Units lyophilized powder for reconstitution only with sterile, preservative-free 0.9% Sodium Chloride Injection, USP, prior to injection.

*Storage :* Unopened vials of XEOMIN can be stored at room temperature 20 to 25°C (68 to 77° F), in a refrigerator at 2 to 8°C (36 to 46°F), or a freezer at -20 to -10°C (-4 to 14°F) for up to 36 months. Do not use after the expiration date on the vial. Reconstituted XEOMIN should be stored in a refrigerator at 2 to 8°C (36 to 46°F) and administered within 24 hours.

*Safe* *Handling*: XEOMIN should not be used if the reconstituted solution has a cloudy appearance or contains floccular or particulate matter. Any reconstituted toxin solution for injection that has been stored for more than 24 hours, as well as any unused solution for injection, should be discarded.

**Dosage Regimen**

100-300 units injected intramuscularly every 16 weeks.

*Rationale*: In clinic, injections are given every 12 weeks. To ensure complete wearing-off of drug effects before re-injection and prevent possible excess weakness, we have extended this period by 4 weeks.

*Drug Schedule:* Any changes to injection dosing or parameters will be based on kinematic measurements, though injection parameters will also be influenced by clinical outcome measures, as is the current standard clinical practice for Xeomin® injections.

**Pre-Study Screening and Baseline Evaluation**

Patients are recruited by the principle investigator from his Movement Disorders Clinic at University Hospital, London, ON. Before enrolling the patient in the study, the patient’s chart is examined to ensure validity for participation. No kinematic assessment, however, is carried out until the participants’ first study visits.

**Treatment / Assessment Visits**

*Figure 1 Time course for the study* *Figure 2 Study Overview*


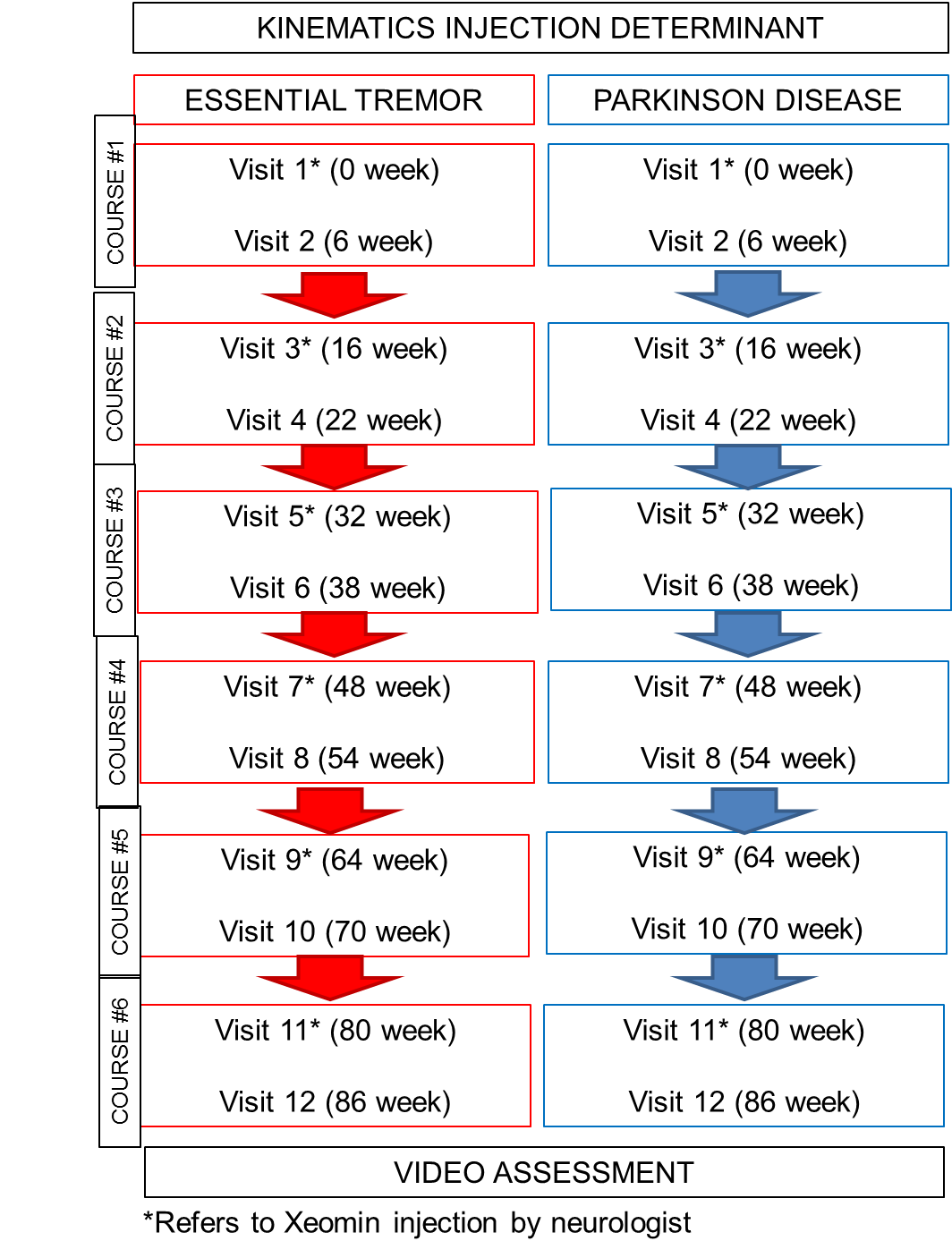


Participants will be fitted with a hospital gown in order to allow full visibility of the limb under study. Participants are seated upright in a straight back chair with armrests. The tremor dominant arm is positioned on a supported armrest. A digital video recording devices is statically positioned to capture a frontal view of the participant from the neck down. The laptop is positioned such that kinematic data is not observable to the participant during the study to avoid a bias or biofeedback effect.

Seven kinematic sensors (Noraxon, INC.) are used to collect data. An electrogoniometer with two degrees of freedom (DOF) is placed at the wrist to collect joint flexion-extension and ulnar-radial deviation information. Another electrogoniometer is placed at the elbow to measure flexion-extension movement and a final electrogoniometer is placed on the shoulder to measure flexion-extension and abduction-adduction movement information. A torsiometer is placed on the underside of the forearm to collect wrist joint pronation-supination deviation information. Three tri-axial accelerometers are placed as follows: one at the distant joint of the third metacarpal, one on the back of the hand and one mid-forearm. The placement of these devices is standardized for each participant. Data recorded from the sensors is wirelessly transmitted to a signal receiver and laptop computer.

Study visits begin with a calibration stage. Calibration is used to determine baseline sensor location, as well as to confirm accuracy of sensor range for each degree of freedom (DOF). Each calibration position is maintained for 5 seconds. Each experimental task is repeated for three trials. Each trial position is maintained for duration of 20 seconds. A 30 second rest period is provided between each trial. Participants are instructed not to speak during recording to avoid confounding tremor data from accessory movement. The calibration and experimental tasks are described in Table 1.

*Table 1. Calibration and Experimental tasks*

| Calibration Tasks | |
| --- | --- |
| 1) 30° Flexion | |
| 2) 30° Extension | |
| 3) 30° Pronation | |
| 4) 30° Supination | |
| 5) 20° Ulnar deviation | |
| 6) 20° Radial deviation | |
| Trial Tasks | |
| 1) Supported Rest | Participants rest their forearm in neutral position on a supportive armrest at mid-trunk height level |
| 2) Pronated Posture | Participants fully extend their arms forward with hands in pronation at shoulder height level |
| 3) Neutral Posture | Participants fully extend their arms forward with thumbs pointing upward at shoulder height level |
| 4) Kinetic | Participants alternate touching fingertip to tip of a pen (held at arm’s reach by a research associate) and their own nose |
| 5) Water Pouring | Participants transfer water between two cups, one in each hand, keeping the transferring hand dynamic and the catching hand static. Distance between hands will be constant across trials |
| 6) Drinking | Participant will take a full cup of water and move the cup to mouth as if drinking |
| 7) Drawing | Participants complete a variety of drawing tasks including Archimedes spirals and connecting two dots right-to-left and left-to-right on an electronic drawing tablet |

Non-kinematic outcome measures are collected at each visit. These include: measures of hand/finger strength to evaluate weakness secondary to Xeomin® injection (Manual Motor Testing and Excess Weakness Scale), measures of quality of life (QUEST), of motor function (Unified Parkinson Disease Rating Scale) and of tremor severity (Fahn-Tolosa-Marin Tremor Rating Scale).

**Concomitant Medication**

*Concomitant medication:* Concomitant treatment of Xeomin® and aminoglycoside antibiotics, spectinomycin, or other agents that interfere with neuromuscular transmission (e.g., tubocurarine-like agents), or muscle relaxants, should be observed closely because the effect of Xeomin® may be potentiated.

*Drug interactions:* No formal drug interaction studies have been conducted with Xeomin®. Use of anticholinergic drugs after administration of Xeomin® may potentiate systemic anticholinergic effects. The effect of administering different botulinum toxin products at the same time or within several months of each other is unknown. Excessive neuromuscular weakness may be exacerbated by administration of another botulinum toxin prior to the resolution of the effects of a previously administered botulinum toxin. Excessive weakness may also be exaggerated by administration of a muscle relaxant before or after administration of Xeomin®.

*Contraindications:* Use in patients with a known hypersensitivity to the active substance botulinum neurotoxin type A, or to any of the excipients (human albumin, sucrose), could lead to a life-threatening allergic reaction. Xeomin® is contraindicated in patients with known hypersensitivity to any botulinum toxin preparation or to any of the components in the formulation. Use in patients with an infection at the injection site could lead to severe local or disseminated infection. Xeomin® is contraindicated in the presence of infection at the proposed injection site(s).

**Rescue Medication & Risk Management**

*Rescue Medication*: Unfortunately, there is no rescue medicine able to reverse the effects of Xeomin®. However, the effects of the injection wear off in 2-3 months, though side effects such as weakness may wear off in as little as 2-3 weeks.

*Immunogenicity*: As with all therapeutic proteins, there is a potential for immunogenicity. The incidence of antibody formation is highly dependent on the sensitivity and specificity of the assay. In addition, the observed incidence of antibody positivity in an assay may be influenced by several factors including assay methodology, sample handling, timing of sample collection, concomitant medications, and underlying disease. For these reasons, comparison of the incidence of antibodies across products in this class may be misleading.

**Premature Withdrawal / Discontinuation Criteria**

1. Withdrawal by Principle Investigator (PI) if PI feels continued participation would impair the participant’s wellbeing.
2. Voluntary withdrawal by subject without prejudice to future treatment by the physician.

**Efficacy Variables and Analysis**

*Clinical efficacy variables (primary endpoint):* Improvement in hand tremor as determined by a decrease > 8 points on a standardized clinical assessment tool (Fahn-Tolosa-Marin Tremor Rating Scale) post-Xeomin® injection. A point change of 8 points was selected based on studies of oral tremor medications showing mean improvement ranging from 7.5 to 10 points increase on the same clinical scale, which statistically produced moderate-large effect sizes.

*Kinematic efficacy variables (secondary endpoint):* Improvement in hand tremor measured as a 50% reduction in overall tremor amplitude post-Xeomin® injection.

**Safety Variables and Analysis**

Any adverse reactions or events are handled in accordance with the University of Western Ontario Health Sciences Research Ethics Board’s (HSREB) policy: patients are encouraged to seek emergency medical care if they feel they are experiencing an adverse reaction. We follow up with this patient in-hospital (if admitted) and after the incident. If the principle investigator feels that the health and/or safety of the patient would be compromised by continuing to participate in the study, the participant will be withdrawn. Any and all adverse events are reported to the HSREB and Merz Pharma Canada Ltd. (supplier of study drug).

**Statistical Analysis**

Flow charts for description of statistical method by research question follow. Alpha level set at 0.05 for all analyses. Statistical method assumes that criteria will be met for parametric statistics.

*Figure 1 (primary endpoint)* - Research Question 1: Is there a significant improvement in tremor severity as measured by standardized clinical assessment tools post Xeomin® injection using kinematic guided injection parameters for both IPD and ET?

Repeated measures ANCOVA was selected to reduce variance associated with baseline variability in tremor severity. This is important for the design given that there is classically significant variability of tremor at baseline making it unrealistic to achieve a rigorously matched sample across all relevant variables.

*Figure 2 (secondary endpoint)* - Research Question 2: Is there a significant reduction in overall tremor amplitude post Xeomin® injection using kinematic guided injection parameters for both IPD and ET?

*Figure 3 (not a clinical endpoint)* - Research Question 3: What is the difference in composition of tremor at baseline between two subgroups of participants: those with > 8 points change and those with ≤ 8 points change on standardized tremor clinical rating scales?
